# Knowledge, Attitudes, and Intentions towards HIV Pre-Exposure Prophylaxis among Nursing Students in Spain

**DOI:** 10.3390/ijerph17197151

**Published:** 2020-09-29

**Authors:** Guillermo López-Díaz, Almudena Rodríguez-Fernández, Eva María Domínguez-Martís, Diego Gabriel Mosteiro-Miguéns, David López-Ares, Silvia Novío

**Affiliations:** 1 Galician Public Health Care Service, University Hospital Complex of Santiago de Compostela (CHUS), 15706 A Coruña, Spain; guilleloopez98@gmail.com (G.L.-D.); diegomoste@gmail.com (D.G.M.-M.); 2 Department of Psiquiatry, Radiology, Public Health, Nursing and Medicine, University of Santiago de Compostela, 15782 A Coruña, Spain; almudena.rodriguez@usc.es; 3 Galician Public Health Care Service, Health Care Centre of Concepción Arenal, C/Santiago León de Caracas 12, 15701 A Coruña, Spain; eva.dominguez2@hotmail.com; 4 Galician Public Health Care Service, University Hospital Complex of A Coruña (CHUAC), 15006 A Coruña, Spain; dlopares@gmail.com

**Keywords:** attitude, HIV, intention, knowledge, nursing, pre-exposure prophylaxis

## Abstract

Human immunodeficiency virus (HIV) infection is one of the main causes of morbidity and mortality worldwide. Among the preventive approaches proposed to control this disease is pre-exposure prophylaxis (PrEP), whose effectiveness depends on the medication adherence. The aim of the present study was to determine the knowledge and attitudes about PrEP among a sample of Spanish nursing students as well as their intentions of receiving it in case it was indicated. An observational cross-sectional descriptive study was carried out. A total of 570 nursing students from the University of Santiago de Compostela (Spain), ≥18 years old and of both sexes were invited to self-complete a questionnaire between February and March 2020. A total of 352 students decided to participate in the study. Participants had low knowledge [overall knowledge score 1(0–2)] and a neutral attitude towards PrEP. The intention of receiving PrEP improved significantly after the completion of the questionnaire and the administration of information about PrEP (*p* = 0.039; before: 23.58% and after: 93.77%). Nursing staff play an important role in the prevention of sexually transmitted diseases, so their training in preventive strategies, such as PrEP, could help to reduce the incidence of new cases of HIV infection.

## 1. Introduction

Acquired immunodeficiency syndrome (AIDS) remains one of the sexually transmitted diseases (STDs) with high incidence and important associated morbidity and mortality [1]. In Spain, with a rate of new HIV diagnoses higher than the average in European Union (EU)/European Economic Area (EEA) [2], a total of 3244 new cases of HIV infection were recorded in 2019, the majority in young men (85.3%, average median age of 36), mainly in men who have sex with men (MSM; 56.4%) [3]. These figures reveal transmission through unprotected sex as the main route of virus spread, and they demonstrate the need for more preventive strategies in combination with classic interventions (such as condom use), which are clearly insufficient [4,5].

Among the approaches proposed, biomedical strategies, and specifically the daily oral antiretroviral pre-exposure prophylaxis (PrEP) has been demonstrated to reduce the risk of transmission in people exposed to but not infected by HIV [6,7,8,9,10], which has lead to that the World Health Organization (WHO) [11] and the Centers for Disease Control and Prevention (CDC) [12] approved its use as preventive measure of HIV transmission. Likewise, as a result of this, the AIDS Study Group (GeSIDA) of the Spanish Society of Infectious Diseases and Clinical Microbiology (SEIMC) developed a programme to support the implementation of PrEP in combination with other preventive measures against HIV infection [9].

The efficacy of daily PrEP based on regimens with tenofovir disoproxil fumarate alone [8] or in combination with emtricitabine (Truvada^®^) [6,13] is strongly dependent on the adherence [5,14]. According to the literature, some of the barriers that could hamper the adherence are: mental health problems, mobility, stigma, risk misperception, the need to conceal PrEP use, social factors (e.g., unstable housing), substance use, cost or the potential side effects [15,16,17]. Although PrEP has not been associated with severe side effects (e.g., gastrointestinal symptoms, nausea, headache, loss of appetite or tiredness), while taking PrEP, a regular clinical and analytical follow-up every 3 months is required in order to evaluate tolerance, toxicity, adherence, HIV infection and other STDs [5,18].

Currently, there still remains a great debate about who could benefit from PrEP. International organizations such as the CDC recommend it for MSM, heterosexually active men and women who are at substantial risk of HIV acquisition, injection drug users as well as serodiscordant couples [12]. On the other hand, the WHO advises the use of PrEP for populations with an HIV incidence of about 3 per 100 person-years or higher [11]. However, despite these recommendations, the difficulty in identifying the target population constitutes a barrier for the prescription of PrEP, along with problems derived from the lack of knowledge of PrEP among providers or the difficulty that patients have in reaching them [19].

Behavioral and biomedical interventions for people who have a substantial risk of HIV acquisition in order to enhance adherence through health education, require continuous follow-up and reiterated preventive advice [20]. Healthcare professionals are responsible for their development, among which include nursing staff. In Spain, a bachelor degree of science in nursing takes four years to complete. As part of the educational preparation, nursing students undertake clinical placements in primary healthcare and hospital settings, and they can work in these settings upon successful graduation. In both settings, students have to follow up on patients with STDs, just as they have to participate in STDs prevention programs, like HIV ones [21]. According to this, the training of future nurses could have a significant impact on the incidence of infectious diseases [22]. To date, there are no studies that have analyzed the level of knowledge, attitudes and intentions in a key professional group in HIV prevention such as nurses, regarding to a relatively novel therapy such as HIV pre-exposure prophylaxis. Thus, the objective of this study was to determine the knowledge and attitudes about PrEP among a sample of Spanish nursing students as well as their intentions of receiving it in case it was indicated.

## 2. Materials and Methods

### 2.1. Design

A cross-sectional, descriptive, observational study was carried out.

### 2.2. Setting and Participants

All nursing students of the University of Santiago de Compostela (USC, Galicia, Spain), one of the three public universities of Galicia, were invited to participate in the study. The investigation included students enrolled in a nursing degree academic course between 2019 and 2020, of either sex and 18 years or older, who voluntarily agreed to participate. Non-Spanish students were excluded from the study.

The size of the study population was 570 at the time of the research. Keeping the expected frequency of all variables at 50%, the desirable sample size using a 95% confidence interval came out to be 307. However, after 15% inflation and rounding, the final desired sample size was determined to be 352.

### 2.3. Questionnaire Design and Data Collection

The questionnaire was designed, according to the advice of healthcare professionals, from the literature review [6,7,8] and previously designed questionnaires [18]. A pilot study was conducted with 10 students, who did not participate in the final study, in order to evaluate the clarity and ease of understanding of the items as well as the filling time of the questionnaire. They reported full comprehension of the questions and ease in completing the questionnaire, so only minimal changes were made following the pilot study.

The questionnaire consists of 37 items structured into five sections (Supplementary Materials, Table S2). The first section includes 10 items about sociodemographic characteristics (age and sex) and other personal data, such as favourite practice area to develop the professional activity; the second section, consisted of 8 closed-ended questions, assesses knowledge about PrEP; the third section measures students’ attitudes regarding PrEP using 15 questions with a five-point Likert scale for each (1 = strongly disagree to 5 = strongly agree). The fourth section includes three closed-ended questions in order to assess student interest in PrEP. After this section, students are given information about PrEP before they can proceed to the last section, consisted of only one closed-ended question, that determines the intention of the student to use PrEP if he/she was a person at substantial risk of HIV infection.

The results regarding knowledge were dichotomized as “correct” or “incorrect” by grouping the answer options as shown in Table S3. For example, for question 11 (the antiretroviral drugs used for PrEP approved by the FDA are), the option “Emtricitabine + tenofovir (Truvada^®^)” was considered “correct,” and the options “Lopinavir (Kaletra^®^)”, “Emtricitabine + tenofovir + efavirenz (Atripla^®^)” and “Do not know/no opinion” were merged as “incorrect”. Then, the variable overall knowledge score (OKS) was estimated for each participant by calculating the proportion of correct answers for the 8 knowledge-based questions and representing this on a scale 0-10 (0: poor knowledge; 10: good knowledge) [23].

In order to assess the reproducibility, 15 students who did not participate in the final study, filled in the questionnaire twice at an interval of 15 days between them.

The questionnaires were anonymous and self-completed between February and March of 2020. Students were free to omit any questions they did not want to answer. No incentive was offered for completing the questionnaire. The questionnaires were distributed by one of the researchers (G.L.D.) using two methods: (i) distribution of the questionnaires in person, which were filled in in the break between classes and left on a desk once they were filled in; (ii) mailing of the questionnaires via the application “Google Surveys”.

### 2.4. Ethical and Legal Considerations

The study was performed with the approval of the Faculty of Nursing, University of Santiago de Compostela (28 January 2020). Likewise, after explaining the procedure and the objective of the investigation, we obtained the students’ consent that their participation was completely voluntary. Pursuant to the Declaration of Helsinki and Data Protection Act (Organic Law 3/2018), data confidentiality was guaranteed at all times.

### 2.5. Statistical Analysis

The results were presented as number and percentage, mean and standard deviation, or median and interquartile range. Numerical (Kolmogorov–Smirnov test, skewness, kurtosis, relationship between mean, median and mode) and visual (plot Q-Q) methods were used to test the normality of data.

Bivariate analysis was performed using Kruskal–Wallis, Mann–Whitney U, ANOVA, Student’s t-tests for continuous variables and chi-square tests for categorical variables. Significance between multiple experimental groups was determined using Tukey’s post hoc analysis. Comparisons were done with t-student test and ANOVA when variables were normally distributed. On the contrary, Mann–Whitney U and Kruskal–Wallis tests were used when variables were not normally distributed. The correlation between attitudes towards PrEP (questions 19-33) and the OKS was studied by Spearman coefficient. Furthermore, bivariate logistic regression was used to identify factors associated with attitudes about PrEP.

Validation of the questionnaire involved analysing its reliability (internal consistency and reproducibility) and validity. The Cronbach α coefficient was calculated for the knowledge section (second section) and test–retest reliability was assessed using the kappa statistic. The Cronbach value for the knowledge domain was 0.80, indicating good internal consistency. Regarding the test–retest analysis (*n* = 15), the global kappa was very good: 0.82 (95% confidence interval 0.794–0.839). On the other hand, content validity was demonstrated since the questionnaire was based on expert consensus.

A *p*-value less than 0.05 was considered significant throughout the study. The softwares GNU PSPP 0.8.4 (Free Software Foundation Inc., Boston, MA, USA) and Epidat version 4.2 (Xunta de Galicia, Santiago de Compostela, Spain) were used for the statistical processing of the data.

## 3. Results

### 3.1. Description of Sample

A total of 570 students of the Degree of Nursing from the University of Santiago de Compostela (157 of the first year, 120 of the second year, 139 of the third year and 154 of the fourth year) were invited to participate in the study, with a response rate of 61.7% (83.4% of the first year, 80% of the second year, 51% of the third year and 35% of the fourth year). The remaining students did not answer the questionnaire online or they were absent from class the day the questionnaire was administered. All students who filled in the questionnaires answered all the questions. Likewise, all students who accessed the online questionnaire sent their answers (bounce rate 0%).

Table 1 shows the sociodemographic characteristics and other personal data of the participants. The sample was composed primarily of women (87.5%) with a median age of 20 years. The students’ favourite practice area to develop their professional activity was, in order of frequency: Emergency, Intensive Care Unit (ICU) and Reanimation Unit (RU); Pediatric nursing and Obstetric-gynecologic nursing.

The students had very little previous training in HIV (38.6%) and only a few of them (14.7%) had ever heard about PrEP, mainly through social networkings, training programme in the nursing degree and traditional communication media. In general, the students were reluctant to ask, as healthcare professionals, about patient sexual orientation (13.9%), although they wouldn’t mind discussing about their sexual risk behaviors (85.8%).

### 3.2. Knowledge of PrEP

The answers to questions about students’ knowledge of PrEP (questions 11-18) are shown in Table 2. In general, knowledge of PrEP was insufficient (OKS= 1(0–2)). The worst results (less than 10% of correct answers) were demonstrated by the participants answering the questions Q11 (drugs used for PrEP), Q14 (contraindications to PrEP use) and Q17 (follow-up visits for patients taking PrEP). On the contrary, the best results (more than 25% of correct answers) were those in relation to requirements before starting PrEP (questions 15 and 16).

Significant differences were found according to: the age (students who were aged ≥ 20 years had a better level of knowledge); the sex (male participants had a better level of knowledge); the class year (students in the last years of their degree had a better level of knowledge); and the prior training on PrEP (students who had ever heard of PrEP had a better level of knowledge). However, no statistically significant differences were found according to the prior training on HIV/AIDS (Table 2).

Knowledge of PrEP, evaluated by the OKS, was significantly higher for students in the last years of their degree (*p* = 0.008) as well as who knew about the existence of the PrEP (*p* = 0.047). However, no statistically significant differences were found according to the sex (*p* = 0.283), age (*p* = 0.409) or prior training on HIV/AIDS (*p* = 0.614) (Table S4).

### 3.3. Attitudes Towards PrEP

The answers to questions about students’ attitudes towards PrEP (questions 19–33) are shown in Table 3. In spite of the fact ~90% of students agreed that nursing staff play an important role in prevention of sexual risk behaviors and STDs through patient education (question 27); it is worth mentioning that they had a neutral opinion for nearly all the items included to know their attitudes towards PrEP. In this sense, >50% of the participants showed a great indifference towards the evidence for PrEP efficacy (questions 19 and 20; 71.3% and 65.3%, respectively), the preference for PrEP over other preventive strategies (question 23; 57.7%), the chance that widespread use of PrEP could increase rates of antiretroviral resistance (question 24; 65.9%), the side effects of PrEP (question 25; 85.8%), the importance of medication adherence (question 26; 62.2%), and the potential barriers to PrEP prescription (question 32; 54–63.9%).

One-third of participants thought that PrEP could cause people to have more risky sex (question 21) and that PrEP is not effective for people with very high risk of HIV infection (question 22). However, the majority of the nursing students identified to serodiscordant couples (66.7%), sex workers (63%), people with multiple sex partners (62.2%) and people with history of STD (60%) as the main groups that could benefit from the PrEP use. When students were asked about why PrEP demand was not higher, they supported the four reasons that were suggested (PrEP or its efficacy is not known, people are ignorant of where PrEP can be obtained and people using PrEP could develop a stigma).

Finally, the majority of the nursing students agreed with allocating resources for PrEP research (question 28) as well as PrEP should be financed by the Social Security System instead of by the patient (questions 29 and 30).

In an analysis that included stratification according to sex, age, number of years of training in the nursing degree and knowledge of PrEP (evaluated by the OKS), the main differences were observed according to the number of years of training in the nursing degree and the knowledge level of PrEP. Concretely, significant differences were found according to: the sex (women disagreed more than men did); the age (students who were aged <20 years disagreed more than students ≥ 20 years); the class year (students in the last years of their degree disagreed more than students in the first years of their degree, except for the questions 19, 20, 26 and 32e for which students in the last years of their degree agreed than students in the first years of their degree); knowledge of PrEP (students with the highest overall knowledge scores agreed more than students with low scores, except for the questions 31c-h for which students with the highest overall knowledge scores disagreed more than students with low scores) (Table 3). However, when bivariate logistic regression was undertaken with the variable “good attitude” set as the dependent variable, almost no significant differences were observed (Table S5).

### 3.4. Interest in PrEP

Although only 38.6% had done some research or had received training on PrEP, and that only 14.7% had ever heard of PrEP, a high percentage of the nursing students (92%) thought it would be “interesting” or “very interesting” to have more PrEP education. Furthermore, their interest was significantly higher among students in the last years of their degree (*p* = 0.018). In relation to the students’preferences for future educational training on PrEP, the most preferred modalities for receiving PrEP education were: subject of the training programme in the nursing degree (mainly during the second or third year), clinical practice guidelines and online methods.

### 3.5. Intention of Receiving PrEP

The intention of receiving PrEP improved significantly after the completion of the questionnaire and the administration of information about PrEP (*p* = 0.039; before: 23.58% and after: 93.77%).

## 4. Discussion

The results of the current study reveal an important knowledge deficit, as well as a great indifference towards PrEP among nursing students, probably due to a lack of familiarity with this HIV prevention measure. To our knowledge, this is the first study in evaluating the level of knowledge and attitudes of nursing students towards PrEP as well as their intentions of receiving it in case it was indicated. Due to the need to determine provider knowledge and attitudes prior to the planning of educational strategies for the prevention of HIV with the aim of maximising their impact, the results of this study are of great interest due to the important role nursing staff play in the prevention of STDs like HIV.

The level of knowledge of PrEP (drugs used, route and schedule of administration, contraindications, requirements prior to the initiation of PrEP, follow-up required and efficacy (questions 11–18)) has been lower than that registered in previous studies carried out among students of other healthcare degrees [24] or healthcare professionals [19,25,26,27,28]. These findings corroborate the lack of approach of this topic in the current curriculum of the nursing degree (83.24 % had never heard of PrEP and less than 50% said that they had received information about it during the training programme in the nursing degree), in spite of scientific evidence supports its usefulness in population groups at high risk for HIV infection, such as MSM [6,7,29], heterosexual women and men [8] and injection drug users [9], as long as there exists a good therapeutic adherence [5,14]. The absence of this topic in the current curriculum of the nursing degree could be related to the recent implementation of PrEP [30], after the last renewal of accreditation of the nursing degree of the University of Santiago de Compostela (academic course 2015–2016). The approach of this topic during the nursing degree would be welcomed by the students, since more than 90% of them showed interest in acquiring more knowledge of PrEP (question 34).

The evaluation of nursing students’ attitudes towards PrEP is of vital importance because of the involvement of nursing staff in activities of preventive education (for example, promotion of therapeutic adherence, obtaining samples during the follow-up of patients at substantial risk of HIV acquisition, among other activities) [31]. Despite the students´ great indifference towards the side effects of PrEP (question 25), which contrasts to other studies [32,33], one of the aspects that worried nursing students was the possibility that the use of PrEP could increase antiretroviral resistance (question 24). Nevertheless, the current evidence suggests that the chance of drug resistance is very low, except when PrEP is started during unrecognized, seronegative acute HIV infection [34,35]. This is the reason why it is necessary to have an HIV test before starting PrEP and do a follow-up every three months to monitor for seroconversion [5,18]. Likewise, one-quarter of participants thought that the use of PrEP could be associated with an increase of sexual risk behaviours (question 21) as well as a weakening of the attention of the National Health System towards other important prevention strategies (question 23). However, up until now, there is no conclusive evidence that its use is associated with an increase of risky behaviours [36], in the same way that the WHO [11] and the CDC [12] promote the use of PrEP in combination with other biomedical, therapeutic, behavioral strategies..., never in substitution with the pre-existing measures. The importance of both classical and more modern measures contrasts greatly with the high percentage of students (~40%, question 22) who rejected PrEP, in favor of the use of condoms, for people at highest risk for HIV infection.

The majority of the students (>60%, question 20) did not know if PrEP was a cost effective intervention; however, >50% considered that it should be financed by the Social Security System (question 30). Given the chronic nature of the HIV infection and the costs derived from its treatment and co-morbidities, PreP it is thought to be profitable [37,38]. However, several studies have pointed out that the high cost of PrEP could be a barrier for its prescription [26,27,39]. In Spain, it should not be a problem as PrEP is a hospital dispensing drug whose cost is covered by the government.

Nursing students identified to serodiscordant couples, sex workers, people with multiple sex partners and people with history of STD as the main groups that could benefit from the PrEP use (question 31). On the contrary, they underestimated the risk of MSM, despite the WHOs´ [40] and CDCs´ [12] recommendations, for being one of the groups with a higher incidence in new diagnoses of HIV in Spain as well as in Europe [3,41]. The identification of risk practices is key to reduce its incidence. The fact that only 13.9% of nursing students, as healthcare professionals, were willing to ask about the sexual orientation of their patients (question 8), would hamper the identification of them, and it highlights that the topic of sexual orientation is still taboo [42].

The PrEP delivery is another aspect widely discussed in the literature [26,27,39,41,43]. To date, PrEP has been delivered in Hospital Pharmacy Units [26], Primary Care Clinics [39,43], HIV Patient Care Clinics [27] or STIs Clinics [41]; however there is no consensus about which is the best option. In Spain, PrEP is prescribed by infectious disease specialists, following consultation with the primary care clinician. It is worth noting that the healthy population for whom PrEP is indicated, may not ask for it or may not go to the follow up visits for fear of being seen with an infectious disease specialist, consequently there are serious doubts about the appropriateness of the specialist as only provider [19]. In this sense, ~ 40% of nursing students said that one of the reasons why PrEP demand in populations at risk of HIV may not be higher was that its prescription could stigmatize these people (question 33).

The knowledge and attitudes towards PrEP influence the development of behavioral skills such as confidence or intentions to discuss PrEP with patients. Specifically, the confidence and the intention to advise patients about PrEP improve as healthcare professionals have greater level of knowledge and more positive attitudes towards PrEP [24]. In the same way, in the current study, an improvement in the intention of receiving PrEP in a hypothetical scenario was found, after giving information about PrEP to the students. The effectiveness of nursing staff in enhancing medication adherence among people infected by HIV [44], reflects the feeling that the majority of the nursing students (>90%, question 27) have on the important role that this group of healthcare professionals play in the prevention of STDs like HIV. Concretely, in Spain, nurses are a key element in multidisciplinary team of healthcare professionals that aid PrEP patients, just as much previously to the beginning of the PrEP from primary healthcare setting (e.g., giving information about effectiveness of PrEP) as well as during its administration in hospital settings (e.g., doing the follow-up every three months, advising in the prevention of drug interactions...). Likewise, nursing staff who work in emergency department, the practice area preferred by students of the present study to develop their future professional activity (question 4), could help to identify HIV-negative patients who would benefit from PrEP, as the emergency department is the place to which people ask for post-exposure prophylaxis.

Our study included several limitations. The main limitation was related to the participation. Despite the fact that it was higher than in other studies [24,45], it was decreasing as students advanced from year to year, which could be associated with the rate of class attendance (higher in the first years of the degree; fourth year students are an exception as they are in practicums all year round). Moreover, the health emergency caused by the SARS-CoV-2 pandemic hindered the distribution of the questionnaire in person. Second, as students filled in questionnaires themselves, there may be some self-report bias. Third, the study included nursing students of only one university, which may limit the external validity of our findings. Because of this limitation, additional studies are needed to determine if the results from our study can be generalized to nursing students with other characteristics (e.g., students who are socioeconomically advantaged). Fourth, as the sample was comprised exclusively of students, we assessed attitudes towards PrEP as an indicator of future behaviors. Fifth, confirmatory or exploratory factor analyses of the questionnaire were not carried out in order to check its factorial validity.

## 5. Conclusions

Nursing students have insufficient knowledge of PrEP and a neutral attitude towards PrEP, although their intention of receiving PrEP improves when they are informed about this HIV prevention measure. These findings highlight the need to carry out more educational activities for future nursing students, with the aim of their participation in current HIV prevention campaigns [21] contributes to maximize impact of PrEP on community health. Likewise, more studies carried out in healthcare workers using health behavior models would be necessary to improve the design of health promotion programs for groups at high risk for HIV infection.

## Figures and Tables

**Table 1:** Sociodemographic characteristics and other personal data of the study’s participants.

	All Students	First Year	Second Year	Third Year	Fourth Year
		N = 131	N = 96	N = 71	N = 54
	N = 352	(37.22%)	(27.27%)	(20.17%)	(15.34%)
Age (years), M (SD) and Med (IQR)	20.4 (3.62)	18.7 (1.2)	20.8 (3.7)	21.3 (2.8)	22.7 (2.3)
	20 (19–21)	18 (18–19)	19 (19–20)	20 (20–21)	21 (21–22.3)
Sex, *n* (%)					
Male	44 (12.5)	17 (12.98)	10 (10.42)	10 (14.08)	7 (12.96)
Female	308 (87.5)	114 (87.02)	86 (89.58)	61 (85.92)	47 (87.04)
Practice area considered, *n* (%)					
Community nursing	59 (16.76)	6 (4.58)	18 (18.75)	20 (28.17)	15 (27.78)
Surgical-medical nursing	83 (23.58)	45 (34.35)	20 (20.83)	10 (14.08)	8 (14.81)
Pediatric nursing	132 (37.5)	55 (41.98)	33 (34.38)	27 (38.03)	17 (31.48)
Emergency, ICU and RU	159 (45.17)	60 (45.8)	39 (40.62)	26 (36.62)	34 (62.96)
Obstetric-gynecologic nursing	118 (33.52)	42 (32.06)	33 (34.38)	23 (32.39)	20 (37.04)
Mental health nursing	52 (14.77)	20 (15.27)	10 (10.42)	12 (16.9)	10 (18.52)
Occupational nursing	4 (1.14)	0	1 (1.04)	1 (1.41)	2 (3.7)
Geriatric nursing	16 (4.55)	5 (3.82)	4 (4.17)	5 (7.04)	2 (3.7)
Other	4 (1.14)	2 (1.53)	0	2 (2.82)	0
I have not decided yet	86 (24.43)	37 (28.24)	23 (23.96)	16 (22.54)	10 (18.52)
Have you done any research or received any training on HIV in your nursing degree?, n (%)					
Yes	136 (38.64)	8 (6.11)	65 (67.71)	31 (43.66)	32 (59.26)
No	196 (55.68)	119 (90.84)	27 (28.12)	32 (45.07)	18 (33.33)
DK/NO	20 (5.68)	4 (3.05)	4 (4.17)	8 (11.27)	4 (7.41)
Have you ever heard of PrEP?, *n* (%)					
Yes	52 (14.77)	11 (8.4)	20 (20.83)	9 (12.68)	12 (22.22)
No	293 (83.24)	119 (90.84)	72 (75.00)	60 (84.51)	42 (77.78)
DK/NO	7 (1.99)	1 (0.76)	4 (4.17)	2 (2.82)	0
Source of information about PrEP, *n* (%)					
Training programme in the nursing degree	24 (46.21)	3 (5.77)	11 (21.15)	4 (7.69)	6 (11.54)
Healthcare professionals	11 (21.15)	3 (5.77)	2 (3.85)	3 (5.77)	3 (5.77)
Clinical practice guidelines	6 (11.54)	1 (1.92)	1 (1.92)	2 (3.85)	2 (3.85)
Relatives or friends	7 (13.46)	2 (3.85)	1 (1.92)	3 (5.77)	1 (1.92)
Traditional communication media	15 (28.85)	5 (9.62)	8 (15.38)	1 (1.92)	1 (1.92)
Social networkings	26 (50)	8 (15.38)	8 (15.38)	6 (11.54)	4 (7.69)
Other	7 (13.46)	3 (5.77)	2 (3.85)	1 (1.92)	1 (1.92)
As healthcare professional, would you ask your patients about their sexual orientation?, *n* (%)					
Yes	49 (13.92)	15 (11.45)	15 (15.62)	12 (16.9)	7 (12.96)
No	245 (69.6)	92 (70.23)	66 (68.75)	49 (69.01)	38 (70.37)
DK/NO	58 (16.48)	24 (18.32)	15 (15.62)	10 (14.08)	9 (16.67)
As healthcare professional, would you ask your patients about their sexual risk behaviors?, *n* (%)					
Yes	302 (85.8)	117 (89.31)	78 (81.25)	61 (85.92)	46 (85.19)
No	22 (6.25)	6 (4.58)	7 (7.29)	6 (8.45)	3 (5.56)
DK/NO	28 (7.95)	8 (6.11)	11 (11.46)	4 (5.63)	5 (9.26)
According to your knowledge, if you belonged to a population group at high risk for HIV infection, would you be willing to receive PrEP?, *n* (%)					
Yes	83 (23.58)	25 (19.08)	27 (28.12)	18 (25.35)	13 (24.07)
No	29 (8.24)	12 (9.16)	3 (3.12)	7 (9.86)	7 (12.96)
DK/NO	240 (68.18)	94 (71.76%)	66 (68.75)	46 (64.79)	34 (62.96)

Abbreviations: DK/NO. Do not know/no opinion; HIV. Human immunodeficiency virus; ICU. Intensive Care Unit; IQR. Interquartile range; M. Mean; Med. Median; RU. Reanimation Unit; SD. Standard deviation.

**Table 2:** Knowledge of pre-exposure prophylaxis (PrEP).

	All Students	*p*
	N = 352	
	*n* (%)	
Q11. The antiretroviral drugs used for PrEP approved by the FDA are:		
Lopinavir (Kaletra^®^)	2 (0.57)	<0.05
Emtricitabine + tenofovir (Truvada^®^)	12 (3.41)	
Emtricitabine + tenofovir + efavirenz (Atripla^®^)	9 (2.56)	
DK/NO	329 (93.47)	
Q12. The PrEP is administered by:		
Intravenous route	6 (1.7)	>0.05
Subcutaneous route	3 (0.85)	
Intramuscular route	1 (0.28)	
Oral route	66 (18.75)	
DK/NO	276 (78.41)	
Q13. According to the FDA, the antiretroviral drugs used for PrEP must be taken:		
Daily	44 (12.50)	<0.05
Weekly	2 (0.57)	
Before sexual intercourse	10 (2.84)	
After sexual intercourse	6 (1.70)	
DK/NO	290 (82.39)	
Q14. PrEP is contraindicated in patients with:		
Creatinine clearance below 60 mL/min	10 (2.84)	<0.05
History of myocardial infarction	2 (0.85)	
Hypertension	7 (1.99)	
DK/NO	329 (93.47)	
Q15. Asymptomatic people must have an HIV test before starting PrEP:		
Yes	119 (33.81)	<0.05
No	20 (5.68)	
DK/NO	213 (60.51)	
Q16. STDs must be ruled out before starting PrEP:		
Yes	150 (42.61)	>0.05
No	4 (1.14)	
DK/NO	198 (56.25)	
Q17. While taking PrEP, people must have regular clinical and analytical follow-up visits with the healthcare provider every:		
Week	7 (1.99)	<0.05
Month	23 (6.53)	
3 months	32 (9.09)	
Year	4 (1.14)	
DK/NO	286 (81.25)	
Q18. PrEP reduces the risk of getting HIV and other STDs:		
Yes	25 (7.10)	<0.05
No	48 (13.64)	
DK/NO	279 (79.26)	
Overall knowledge score	1 (0-2)	<0.05

The correct answers have been underlined. The answers were compared according to the sex, age, class year and prior training or knowledge of HIV/AIDS and PrEP. Statistical significance (*p* < 0.05) was determined by chi-square test (questions 11–18) and Kruskal-Wallis and Mann-Whitney U tests (overall knowledge score). Abbreviations: DK/NO. Do not know/no opinion; FDA. Food and Drug Administration; HIV. Human immunodeficiency virus; PrEP. Pre-exposure prophylaxis; Q. Question; STDs. Sexually transmitted diseases.

**Table 3:** Attitudes towards pre-exposure prophylaxis (PrEP).

	Total						Mean (SD)	*p*
	N = 352							
	*n* (%)							
	1	2	3	4		5		
Q19. There is insufficient evidence at this time to consider PrEP an appropriate prevention strategy	17 (4.83)	35 (9.94)	251 (71.31)	40 (11.36)		9(2.56)	2.97 (0.71)	<0.05
Q20. PrEP is a cost-effective HIV prevention intervention if used with an appropriate population of patients	2 (0.57)	11 (3.12)	230 (65.34)	93 (26.42)		16 (4.55)	3.31 (0.64)	<0.05
Q21. PrEP will cause people to have more risky sex	19 (5.4)	75 (21.31)	148 (42.05)	91 (25.85)		19(5.4)	3.05 (0.95)	<0.05
Q22. People with very high risk of HIV infection must be encouraged to use condoms rather than to take PrEP	7 (1.99)	35 (9.94)	170 (48.3)	91 (25.85)		49 (13.92)	3.4 (0.92)	>0.05
Q23. PrEP may be given preference over other preventive strategies	16 (4.55)	69 (19.6)	203 (57.67)	57 (16.19)		7(1.99)	2.91 (0.78)	>0.05
Q24. Widespread use of PrEP will likely significantly increase rates of antiretroviral resistance	2 (0.57)	23 (6.53)	232 (65.91)	82 (23.3)		13 (3.69)	3.23 (0.65)	>0.05
Q25. PrEP is associated to important side effects	3 (0.85)	24 (6.82)	302 (85.8)	19 (5.4)		4(1.14)	2.99 (0.45)	<0.05
Q26. PrEP adherence is critical to efficacy	2 (0.57)	8(2.27)	219 (62.22)	83 (23.58)		40 (11.36)	3.43 (0.74)	<0.05
Q27. Nurses play an important role in prevention of sexual risk behaviors and STDs, such as HIV, through patient education	2 (0.57)	3 (0.85)	33 (9.38)	52 (14.77)		262 (74.43)	4.62 (0.74)	>0.05
Q28. It is neccessary to allocate resources for PrEP research	2 (0.57)	4 (1.14)	87 (24.72)	144 (40.91)		115 (32.67)	4.04 (0.82)	<0.05
Q29. PrEP must be paid by the patient	69 (19.6)	110 (31.25)	156 (44.32)	12 (3.41)		5 (1.42)	2.36 (0.88)	>0.05
Q30. PrEP must be financed by the Social Security System	3 (0.85)	12 (3.41)	140 (39.77)	125 (35.51)		72 (20.45)	3.71 (0.86)	>0.05
Q31. PrEP is recommended for:								
a. Men who have sex with men	11 (3.12)	9 (2.56)	134 (38.07)	107 (30.4)		91 (25.85)	3.73 (0.98)	<0.05
b. Heterosexual people	9 (2.56)	12 (3.41)	145 (41.19)	101 (28.69)		85 (24.15)	3.68 (0.96)	<0.05
c. Transgender people	10 (2.84)	10 (2.84)	161 (45.74)	86 (24.43)		85 (24.15)	3.64 (0.97)	<0.05
d. Sex workers	6 (1.7)	9 (2.56)	115 (32.67)	108 (30.68)		114 (32.39)	3.89 (0.95)	<0.05
e. People with multiple sex partners	7 (1.99)	6 (1.7)	120 (34.09)	109 (30.97)		110 (31.25)	3.88 (0.94)	<0.05
f. People with history of STD	4 (1.14)	5 (1.42)	131 (37.22)	100 (28.41)		111 (31.53)	3.88 (0.92)	<0.05
g. Injection drug users	4 (1.14)	6 (1.7)	134 (38.07)	103 (29.26)		105 (29.83)	3.85 (0.91)	<0.05
h. Serodiscordant couples	4 (1.14)	2 (0.57)	111 (31.53)	114 (32.39)		121 (34.38)	3.98 (0.89)	<0.05
Q32. PrEP prescription remains suboptimal because...								
a. It is difficult to identify the target population	4 (1.14)	28 (7.95)	225 (63.92)	73 (20.74)		22 (6.25)	3.23 (0.73)	>0.05
b. Physicians have insufficient knowledge of PrEP	4 (1.14)	12 (3.41)	190 (53.98)	104 (29.55)		42 (11.93)	3.48 (0.79)	>0.05
c. There is lack of time to follow-up the patients	1 (0.28)	19 (5.4)	203 (57.67)	91 (25.85)		38 (10.8)	3.41 (0.77)	<0.05
d. There is lack of time to advise on the prevention of sexual risk behaviors	6 (1.7)	27 (7.67)	196 (55.68)	84 (23.86)		39 (11.08)	3.35 (0.84)	>0.05
e. There are not protocols or clinical practice guidelines	2 (0.57)	18 (5.11)	202 (57.39)	84 (23.86)		46 (13.07)	3.44 (0.8)	<0.05
f. Healthcare professionals do not discuss sexual risk behaviors with their patients	4 (1.14)	15 (4.26)	207 (58.81)	85 (24.15)		41 (11.65)	3.41 (0.79)	>0.05
Q33. PrEP demand is not higher because…								
a. Its existence is unknown	4 (1.14)	3 (0.85)	106 (30.11)	113 (32.1)		126 (35.8)	4.01 (0.89)	>0.05
b. People do not know where it can be obtained	1 (0.28)	6 (1.7)	135 (38.35)	104 (29.55)		106 (30.11)	3.88 (0.88)	>0.05
c. It could stigmatize people	1 (0.28)	10 (2.84)	165 (46.88)	98 (27.84)		78 (22.16)	3.69 (0.86)	<0.05
d. People do not know its efficacy	1 (0.28)	6 (1.7)	137 (38.92)	112 (31.82)		96 (27.27)	3.84 (0.86)	>0.05

Answers were expressed on a five-point Likert scale (1 = strongly disagree to 5 = strongly agree). Statistical significance (*p* < 0.05) was determined by ANOVA and Student’s t-tests and Spearman’s correlation analysis. Abbreviations: HIV. Human immunodeficiency virus; PrEP. Pre-exposure prophylaxis; Q. Question; SD. Standard deviation; STDs. Sexually transmitted diseases.

## Supplementary Materials

The following are available online at https://www.mdpi.com/1660-4601/17/19/7151/s1, Table S1: Cuestionario sobre conocimientos, actitudes e intenciones hacia la profilaxis preexposición (PrEP) al VIH. Table S2. English version of the questionnaire “knowledge, attitudes, and intentions towards HIV pre-exposure prophylaxis (PrEP). Table S3: Recoding into two categories (correct/incorrect) of the answers to the questions included in the knowledge section of the questionnaire. Table S4: Overall knowledge score according to the sex, age, class year and prior training or knowledge of HIV/AIDS and PrEP. Table S5: Bivariate logistic regression analysis for attitudes about PrEP.

## References

[1] UNAIDS Ending the AIDS Epidemic. https://www.unaids.org/sites/default/files/20200706_PR_Global_Report_en.pdf.

[2] World Health Organization HIV/AIDS Surveillance in Europe. https://www.ecdc.europa.eu/sites/default/files/documents/hiv-surveillance-report-2019.pdf.

[3] Unidad de Vigilancia de VIH y Comportamientos de Riesgo Vigilancia Epidemiológica del VIH y Sida en España 2018: Sistema de Información Sobre Nuevos Diagnósticos de VIH y Registro Nacional de Casos de Sida. https://www.mscbs.gob.es/ciudadanos/enfLesiones/enfTransmisibles/sida/vigilancia/doc/Informe_VIH_SIDA_2019_21112019.pdf.

[4] Rivero A., Moreno S. (2017). Is it time to start new HIV prevention strategies in Spain?. Enferm. Infecc. Microbiol. Clin..

[5] Moreno S., Antela A., García F., Del Amo J., Boix V., Coll P., Fortuny C., Sirvent J., Gutiérrez F., Iribarren J.A. (2017). Executive summary: Pre-exposure prophylaxis for prevention of HIV infection in adults in Spain: July 2016. Enferm. Infecc. Microbiol. Clin..

[6] McCormack S., Dunn D.T., Desai M., Dolling D.I., Gafos M., Gilson R., Sullivan A.K., Clarke A., Reeves I., Schembri G. (2016). Pre-exposure prophylaxis to prevent the acquisition of HIV-1 infection (PROUD): Effectiveness results from the pilot phase of a pragmatic open-label randomised trial. Lancet.

[7] Grant R.M., Lama J.R., Anderson P.L., McMahan V., Liu A.Y., Vargas L., Goicochea P., Casapía M., Guanira-Carranza J.V., Ramirez-Cardich M.E. (2010). Preexposure chemoprophylaxis for HIV prevention in men who have sex with men. N. Engl. J. Med..

[8] Baeten J.M., Donnell D., Ndase P., Mugo N.R., Campbell J.D., Wangisi J., Tappero J.W., Bukusi E.A., Cohen C.R., Katabira E. (2012). Antiretroviral prophylaxis for HIV prevention in heterosexual men and women. N. Engl. J. Med..

[9] Choopanya K., Martin M., Suntharasamai P., Sangkum U., Mock P.A., Leethochawalit M., Chiamwongpaet S., Kitisin P., Natrujirote P., Kittimunkong S. (2013). Antiretroviral prophylaxis for HIV infection in injecting drug users in Bangkok, Thailand (the Bangkok Tenofovir Study): A randomised, double-blind, placebo-controlled phase 3 trial. Lancet.

[10] Ming K., Shrestha I., Vazquez A., Wendelborn J., Jimenez V., Lisha N., Neilands T.B., Scott H., Liu A., Steward W. (2020). Improving the HIV PrEP continuum of care using an intervention for healthcare providers: A stepped-wedge study protocol. BMJ Open.

[11] World Health Organization WHO Expands Recommendation on Oral Pre-Exposure Prophylaxis of HIV Infection (PrEP). https://www.who.int/hiv/pub/prep/policy-brief-prep-2015/en/.

[12] Centers for Disease Control and Prevention: US Public Health Service Preexposure Prophylaxis for the Prevention of HIV Infection in the United States—2017 Update: A Clinical Practice Guideline. https://www.cdc.gov/hiv/pdf/risk/prep/cdc-hiv-prep-guidelines-2017.pdf.

[13] Centers for Disease Control and Prevention (CDC) (2012). Interim guidance for clinicians considering the use of preexposure prophylaxis for the prevention of HIV infection in heterosexually active adults. MMWR Morb. Mortal. Wkly. Rep..

[14] Wechsberg W.M., Browne F.A., Ndirangu J., Bonner C.P., Minnis A.M., Nyblade L., Speizer I.S., Howard B.N., Myers B., Ahmed K. (2020). The prepare pretoria project: Protocol for a cluster-randomized factorial-design trial to prevent HIV with PrEP among adolescent girls and young women in Tshwane, South Africa. BMC Public Health.

[15] Amico K.R., Marcus J.L., McMahan V., Liu A., Koester K.A., Goicochea P., Anderson P.L., Glidden D., Guanira J., Grant R. (2014). Study product adherence measurement in the iPrEx placebo-controlled trial: Concordance with drug detection. J. Acquir. Immune Defic. Syndr..

[16] van der Straten A., Stadler J., Montgomery E., Hartmann M., Magazi B., Mathebula F., Schwartz K., Laborde N., Soto-Torres L. (2014). Women’s experiences with oral and vaginal pre-exposure prophylaxis: The VOICE-C qualitative study in Johannesburg, South Africa. PLoS ONE.

[17] Scholl E. (2016). Improving outpatient implementation of preexposure prophylaxis in men who have sex with men. J. Am. Assoc. Nurse Pract..

[18] O’Byrne P., MacPherson P., Orser L., Jacob J.D., Holmes D. (2019). PrEP-RN: Clinical considerations and protocols for nurse-led PrEP. J. Assoc. Nurses AIDS Care.

[19] Smith D.K., Mendoza M.C., Stryker J.E., Rose C.E. (2016). PrEP awareness and attitudes in a national survey of primary care clinicians in the United States, 2009–2015. PLoS ONE.

[20] Mayer K.H., Safren S.A., Elsesser S.A., Psaros C., Tinsley J.P., Marzinke M., Clarke W., Hendrix C., Wade Taylor S., Haberer J. (2017). Optimizing pre-exposure antiretroviral prophylaxis adherence in men who have sex with men: Results of a pilot randomized controlled trial of “Life-Steps for PrEP”. AIDS Behav..

[21] Servizo Galego de Saúde Campañas de Prevención del VIH en App Para HSH. https://www.sergas.es/Saude-publica/Campana-2019-app-HSH.

[22] Nelson L.E., McMahon J.M., Leblanc N.M., Braksmajer A., Crean H.F., Smith K., Xue Y. (2019). Advancing the case for nurse practitioner-based models to accelerate scale-up of HIV pre-exposure prophylaxis. J. Clin. Nurs..

[23] Inácio J., Barnes L.M., Jeffs S., Castanheira P., Wiseman M., Inácio S., Bowler L., Lansley A. (2017). Master of pharmacy students’ knowledge and awareness of antibiotic use, resistance and stewardship. Curr. Pharm. Teach. Learn..

[24] Przybyla S.M., Parks K., Bleasdale J., Sawyer J., Morse D. (2019). Awareness, knowledge, and attitudes towards human immunodeficiency virus (HIV) pre-exposure prophylaxis (PrEP) among pharmacy students. Curr. Pharm. Teach. Learn..

[25] Petroll A.E., Walsh J.L., Owczarzak J.L., McAuliffe T.L., Bogart L.M., Kelly J.A. (2017). PrEP awareness, familiarity, comfort, and prescribing experience among US primary care providers and HIV specialists. AIDS Behav..

[26] Ferrández J.S., Sesmero J.M., Aznárez H.N., Espínola S.F., Rodríguez I.E., Cerdá J.V. (2016). Perceptions about HIV pre-exposure prophylaxis among healthcare professionals in Spain (PERPPRES Study). Farm. Hosp..

[27] Blumenthal J., Jain S., Krakower D., Sun X., Young J., Mayer K., Haubrich R., CCTG 598 Team (2015). Knowledge is power! Increased provider knowledge scores regarding pre-exposure prophylaxis (PrEP) are associated with higher rates of PrEP prescription and future intent to prescribe PrEP. AIDS Behav..

[28] Blackstock O.J., Moore B.A., Berkenblit G.V., Calabrese S.K., Cunningham C.O., Fiellin D.A., Patel V.V., Phillips K.A., Tetrault J.M., Shah M. (2017). A cross-sectional online survey of HIV pre-exposure prophylaxis adoption among primary care physicians. J. Gen. Intern. Med..

[29] Molina J.M., Capitant C., Spire B., Pialoux G., Cotte L., Charreau I., Tremblay C., Le Gall J.M., Cua E., Pasquet A. (2015). On-demand preexposure prophylaxis in men at high risk for HIV-1 infection. N. Engl. J. Med..

[30] García L.M., Iniesta C., Garrido J., Fuster M.J., Pujol F., Meulbroek M., Poveda T., Riera M., Antela A., Moreno S. (2019). HIV pre-exposure prophylaxis (PrEP) in Spain: Political and administrative situation. Enferm. Infecc. Microbiol. Clin..

[31] Nokes K.M. (2000). Exploring the clinical nurse specialist role in an AIDS community-based organization. Clin. Nurse Spec..

[32] Puro V., Palummieri A., De Carli G., Piselli P., Ippolito G. (2013). Attitude towards antiretroviral pre-exposure prophylaxis (PrEP) prescription among HIV specialists. BMC Infect. Dis..

[33] Tellalian D., Maznavi K., Bredeek U.F., Hardy W.D. (2013). Pre-exposure prophylaxis (PrEP) for HIV infection: Results of a survey of HIV healthcare providers evaluating their knowledge, attitudes, and prescribing practices. AIDS Patient Care STDs.

[34] Lehman D.A., Baeten J.M., McCoy C.O., Weis J.F., Peterson D., Mbara G., Donnell D., Thomas K.K., Hendrix C.W., Marzinke M.A. (2015). Risk of drug resistance among persons acquiring HIV within a randomized clinical trial of single- or dual-agent preexposure prophylaxis. J. Infect. Dis..

[35] Parikh U.M., Mellors J.W. (2016). Should we fear resistance from tenofovir/emtricitabine preexposure prophylaxis?. Curr. Opin. HIV AIDS.

[36] Freeborn K., Portillo C.J. (2018). Does pre-exposure prophylaxis for HIV prevention in men who have sex with men change risk behaviour? A systematic review. J. Clin. Nurs..

[37] Cambiano V., Miners A., Dunn D., McCormack S., Ong K.J., Gill O.N., Nardone A., Desai M., Field N., Hart G. (2018). Cost-effectiveness of pre-exposure prophylaxis for HIV prevention in men who have sex with men in the UK: A modelling study and health economic evaluation. Lancet Infect. Dis..

[38] Punyacharoensin N., Edmunds W.J., De Angelis D., Delpech V., Hart G., Elford J., Brown A., Gill O.N., White R.G. (2016). Effect of pre-exposure prophylaxis and combination HIV prevention for men who have sex with men in the UK: A mathematical modelling study. Lancet HIV.

[39] Krakower D., Ware N., Mitty J.A., Maloney K., Mayer K.H. (2014). HIV providers’ perceived barriers and facilitators to implementing pre-exposure prophylaxis in care settings: A qualitative study. AIDS Behav..

[40] World Health Organization Policy Brief on Oral Pre-Exposure Prophylaxis of HIV Infection (PrEP). https://apps.who.int/iris/bitstream/handle/10665/197906/WHO_HIV_2015.48_eng.pdf;jsessionid=A6D4B34D47A03C7D157F2830F0B10D36?sequence=1.

[41] Ayerdi-Aguirrebengoa O., Vera-García M., Puerta-López T., Raposo-Utrilla M., Rodríguez-Martín C., Del Romero-Guerrero J. (2017). To whom is HIV pre-exposure prophylaxis proposed?. Enferm. Infecc. Microbiol. Clin..

[42] Fuzzell L., Fedesco H.N., Alexander S.C., Fortenberry J.D., Shields C.G. (2016). “I just think that doctors need to ask more questions”: Sexual minority and majority adolescents’ experiences talking about sexuality with healthcare providers. Patient Educ. Couns..

[43] Thrun M.W. (2014). Opportunity knocks: HIV prevention in primary care. LGBT Health.

[44] Van Camp Y.P., Van Rompaey B., Elseviers M.M. (2013). Nurse-led interventions to enhance adherence to chronic medication: Systematic review and meta-analysis of randomised controlled trials. Eur. J. Clin. Pharmacol..

[45] Calabrese S.K., Earnshaw V.A., Underhill K., Hansen N.B., Dovidio J.F. (2014). The impact of patient race on clinical decisions related to prescribing HIV pre-exposure prophylaxis (PrEP): Assumptions about sexual risk compensation and implications for access. AIDS Behav..

