# Supplementary material for: Knowledge, Attitudes, and Intentions towards HIV Pre-Exposure Prophylaxis among Nursing Students in Spain
Source: Int J Environ Res Public Health. 2020 Sep 29;17(19):7151. doi: 10.3390/ijerph17197151 (PMC7578937; doi:10.3390/ijerph17197151)
Supplement: Supplementary file 1 [file ijerph-17-07151-s001.pdf]

Supplementary Materials

Knowledge, Attitudes, and Intentions towards HIV Pre-Exposure Prophylaxis Among Nursing Students in Spain

Guillermo López-Díaz [1], Almudena Rodríguez-Fernández [2], Eva María Domínguez-Martís [3], Diego Gabriel Mosteiro-Miguéns [1], David López-Ares [4] and Silvia Novío [2,*]

1 Galician Public Health Care Service, University Hospital Complex of Santiago de Compostela (CHUS), 15706 A Coruña, Spain; Guillermo.Lopez.Diaz@sergas.es (G.L.-D.); diegomoste@gmail.com (D.G.M.-M.)
2 Department of Psiquiatry, Radiology, Public Health, Nursing and Medicine, University of Santiago de Compostela, 15782 A Coruña, Spain; almudena.rodriguez@usc.es
3 Galician Public Health Care Service, Health Care Centre of Concepción Arenal, C/Santiago León de Caracas 12, 15701 A Coruña, Spain; eva.dominguez2@hotmail.com
4 Galician Public Health Care Service, University Hospital Complex of A Coruña (CHUAC), 15006 A Coruña, Spain; dlopares@gmail.com
* Correspondence: silvia.novio@usc.es; Tel.: +34-881-812-374.

Table S1. Cuestionario sobre conocimientos, actitudes e intenciones hacia la profilaxis preexposición (PrEP) al VIH.

PRIMERA PARTE: DATOS SOCIODEMOGRÁFICOS

1. Sexo:
   - □ Masculino
   - □ Femenino

2. Edad (en años):_____

3. ¿Qué año del grado en enfermería está usted cursando actualmente?
   □ 1º □ 2º □ 3º □ 4º

4. ¿En qué área de enfermería está considerando trabajar cuando se gradúe? (Por favor, seleccione todas las que considere):
   - □ Enfermería comunitaria.
   - □ Enfermería médico-quirúrgica.
   - □ Enfermería pediátrica.
   - □ Servicios especiales (Urgencias, UCI, REA).
   - □ Enfermería obstétrico-ginecológica.
   - □ Enfermería de la salud mental.
   - □ Enfermería del trabajo.
   - □ Enfermería geriátrica.
   - □ Otras áreas de enfermería. Indique cual: ____________________
   - □ No lo he decidido todavía.

5. ¿Ha hecho alguna investigación o ha recibido algún tipo de formación sobre VIH en el grado en enfermería?
   □ Sí □ No □ No sabe/No contesta

6. Previamente a este cuestionario, ¿había escuchado el término "PrEP" para la prevención del VIH?
   □ Sí □ No □ No sabe/No contesta

7. ¿De qué fuente(s) ha obtenido información sobre PrEP? (Por favor, marque todas las que corresponda):
   - □ Asignatura de mis estudios académicos.
   - □ Información de profesionales sanitarios.
   - □ Guías de práctica clínica.
   - □ Utilizada por algún familiar o amigo.
   - □ Utilizada por algún paciente mientras realizaba prácticas.
   - □ Medios de comunicación tradicionales (prensa, TV, radio).
   - □ Redes sociales.
   - □ Otras fuentes (por favor, indique cual):_____________________

8. Como profesional sanitario, ¿le preguntaría a sus pacientes por su orientación sexual?

□ Sí □ No □ No sabe/No contesta

9. Como profesional sanitario, ¿le preguntaría a sus pacientes si realizan prácticas sexuales de riesgo?

□ Sí □ No □ No sabe/No contesta

10. Con sus conocimientos actuales, ¿estaría dispuesto/a a tomar PrEP?

□ Sí □ No □ No sabe/No contesta

SEGUNDA PARTE: CONOCIMIENTOS GENERALES SOBRE PrEP

Por favor, marque con una "X" la/s respuesta/s que considere correcta/s.

11. Los fármacos antirretrovirales utilizados en la PrEP que están aprobados por la *Food and Drug Administration* (FDA) son:

□ Lopinavir, comercialmente conocido como Kaletra.
□ Emtricitabine + tenofovir, comercialmente conocido como Truvada.
□ Emtricitabine + tenofovir + efavirenz, comercialmente conocido como Atripla.
□ No sabe/No contesta.

12. La vía de administración de la PrEP es:

□ Vía intravenosa.
□ Vía oral.
□ Vía intramuscular.
□ Vía subcutánea.
□ No sabe/No contesta.

13. De acuerdo con la FDA, los fármacos antirretrovirales utilizados en la PrEP deben tomarse:

□ Semanalmente.
□ Diariamente.
□ Previamente a relaciones sexuales.
□ Tras relaciones sexuales.
□ No sabe/No contesta.

14. Le prescripción de la PrEP está contraindicada en pacientes con :

□ Bajo aclaramiento de creatinina (<60 mL/min).
□ Antecedentes de infarto de miocardio.
□ Hipertensión.
□ No sabe/No contesta.

15. Antes de iniciar la PrEP en un paciente asintomático es necesario realizar una prueba de detección del VIH.

□ Sí □ No □ No sabe/No contesta

16. Antes de iniciar la PrEP es recomendable valorar la existencia de otras Enfermedades de Transmisión Sexual (ETS).

□ Sí □ No □ No sabe/No contesta

17. Una vez iniciada la PrEP la frecuencia con que debería hacerse un seguimiento clínico y analítico es:

□ Semanalmente.
□ Mensualmente.
□ Trimestralmente.
□ Anualmente.
□ No sabe/No contesta.

18. La PrEP, además de frente al VIH, ofrece protección frente a otras ETS.

□ Sí □ No □ No sabe/No contesta

TERCERA PARTE: ACTITUDES HACIA LA PrEP

Por favor, elija la opción que mejor refleje su opinión sobre cada uno de los siguientes enunciados.

	Muy en desacuerdo	En desacuerdo	Ninguna opinión	De acuerdo	Totalmente de acuerdo

19. Actualmente existe escasa evidencia para considerar la PrEP como una intervención eficaz para prevenir la transmisión del VIH.					
20. En determinados grupos de población, la PrEP es una intervención costo-efectiva en la prevención del VIH.					
21. El uso de PrEP podría aumentar las conductas sexuales de riesgo.					
22. A las personas con mayor riesgo de adquirir VIH se les debería recomendar el uso de preservativo en vez de PrEP.					
23. La PrEP puede conllevar una disminución en la atención por parte del sistema sanitario sobre otras importantes estrategias de prevención.					
24. El uso extendido de PrEP podría aumentar las tasas de resistencia a antirretrovirales.					
25. La PrEP se asocia a importantes efectos adversos.					
26. La adherencia es un factor clave en la efectividad de la PrEP.					
27. Enfermería juega un papel importante en la educación sanitaria para la					

prevención de conductas sexuales de riesgo y ETS, como el VIH					
28. Deben destinarse recursos a la investigación de PrEP.					
29. La PrEP debería ser costeada por el usuario.					
30. La PrEP debería ser costeada por la Seguridad Social.					
31. La PrEP debe ser recomendada a:					
a. Hombres que mantienen relaciones sexuales con hombres.					
b. Personas que mantienen relaciones sexuales con el sexo opuesto.					
c. Personas transgénero.					
d. Trabajadores/as sexuales.					
e. Personas con múltiples parejas sexuales.					
f. Personas con historial de ETS previas.					
g. Consumidores de drogas por vía intravenosa.					
h. Personas no infectadas con pareja VIH positiva (parejas serodiscordantes).					
32. La prescripción de PrEP no es mayor debido a:					
a. La dificultad para identificar grupos de riesgo.					
b. La falta de conocimientos de los prescriptores.					

c. La falta de tiempo para realizar un seguimiento clínico y analítico.					
d. La falta de tiempo para aconsejar sobre reducción de prácticas de riesgo.					
e. La falta de protocolos/guías de práctica clínica.					
f. La falta de confianza de los prescriptores para preguntar sobre actividades sexuales.					
33. La demanda de PrEP no es mayor entre la población diana porque:					
a. Desconoce su existencia.					
b. Desconoce dónde solicitarla.					
c. No quiere ser estigmatizada.					
d. Desconoce su eficacia.					

CUARTA PARTE: INTERÉS POR LA PrEP

34. ¿Cuál es su nivel de interés en cuanto a la adquisición de más conocimientos sobre PrEP para la prevención del VIH?

- □ Muy desinteresado.
- □ Desinteresado.
- □ Neutral.
- □ Interesado.
- □ Muy interesado.

35. Por favor, conteste solamente si su respuesta anterior fue Interesado/Muy interesado. ¿A través de qué método le gustaría aprender más sobre PrEP? (Por favor, marque todas las que considere).

- □ Libros de texto.
- □ Métodos online.
- □ Asignaturas de mis estudios académicos.
- □ Guías de práctica clínica.
- □ Otros:________________

36. ¿En qué curso cree que el grado de enfermería debería invertir más tiempo en formar sobre PrEP? (Seleccione todas las que considere):

- □ 1º
- □ 2º
- □ 3º
- □ 4º
- □ Ninguno de los anteriores

QUINTA PARTE: INTENCIONES

Necesita conocer la siguiente información sobre la PrEP antes de continuar:

La PrEP es una intervención preventiva que reduce el riesgo de adquirir el VIH en personas seronegativas con alto riesgo de contraer la infección. Consiste en la toma diaria de un comprimido oral

de un fármaco antirretroviral comercialmente conocido como Truvada® (Tenofovir + Emtricitabina), que manteniendo una correcta adherencia diaria junto con otras estrategias preventivas ha demostrado una alta eficacia en la reducción de la transmisión del VIH antes de su exposición.

Es importante conocer que no protege frente a otras ETS, por lo que se precisan otras medidas de protección como el uso de preservativo en las relaciones sexuales.

Antes de iniciar la PrEP hay que realizar una prueba de detección del VIH, así como de otras posibles ETS; siendo además necesario valorar la función renal previamente a su prescripción. Asimismo, una vez iniciada, las personas con PrEP tienen que hacer un seguimiento clínico y analítico cada tres meses mientras persista su indicación.

Los efectos adversos comunes de la PrEP incluyen: molestias gastrointestinales, náuseas, cefalea, falta de apetito o cansancio. Sin embargo, generalmente se atenúan con el tiempo y no se han observado efectos secundarios graves.

De acuerdo con la información anteriormente facilitada ¿estaría dispuesto/a a utilizar PrEP si fuese una persona susceptible con alto riesgo de adquirir el VIH?
□ Sí<br>□ No<br>□ No sabe/No contesta

Table S2. English version of the questionnaire "knowledge, attitudes, and intentions towards HIV pre-exposure prophylaxis (PrEP)

FIRST SECTION: SOCIODEMOGRAPHIC CHARACTERISTICS

1. Sex:
   - □ Male
   - □ Female

2. Age (years):_____

3. What year of nursing degree are you currently in?
   □ 1º □ 2º □ 3º □ 4º

4. Which is your favourite practice area to develop your professional activity? (Please, choose all that apply):
   - □ Community nursing
   - □ Surgical-medical nursing
   - □ Pediatric nursing
   - □ Emergency, ICU and RU
   - □ Obstetric-gynecologic nursing
   - □ Mental health nursing
   - □ Occupational nursing
   - □ Geriatric nursing
   - □ Other: ____________________
   - □ I have not decided yet

5. Have you done any research or received any training on HIV in your nursing degree?
   □ Yes □ No □ Do not know /no opinion

6. Have you ever heard of PrEP?
   □ Yes □ No □ Do not know /no opinion

7. How have you obtained information about PrEP? (Please, choose all that apply):
   - □ Training programme in the nursing degree
   - □ Healthcare professionals
   - □ Clinical practice guidelines
   - □ Relatives or friends
   - □ During clinical placements (taken by patients)
   - □ Traditional communication media
   - □ Social networkings
   - □ Other:_____________________

8. As healthcare professional, would you ask your patients about their sexual orientation?
   □ Yes □ No □ Do not know /no opinion

9. As healthcare professional, would you ask your patients about their sexual risk behaviors?
   □ Yes □ No □ Do not know /no opinion

10. According to your knowledge, if you belonged to a population group at high risk for HIV infection, would you be willing to receive PrEP?
    □ Yes □ No □ Do not know /no opinion

SECOND SECTION: GENERAL KNOWLEDGE ABOUT PrEP

Please mark the correct answers to the questions below.

11. The antiretroviral drugs used for PrEP approved by the Food and Drug Administration (FDA) are:
    - □ Lopinavir (Kaletra®)
    - □ Emtricitabine + tenofovir (Truvada®)
    - □ Emtricitabine + tenofovir + efavirenz (Atripla®)
    - □ Do not know /no opinion

12. The PrEP is administered by:
    - □ Intravenous route
    - □ Oral route
    - □ Intramuscular route
    - □ Subcutaneous route
    - □ Do not know /no opinion

13. According to the FDA, the antiretroviral drugs used for PrEP must be taken:
- □ Weekly
- □ Daily
- □ Before sexual intercourse
- □ After sexual intercourse
- □ Do not know /no opinion

14. PrEP is contraindicated in patients with:
- □ Creatinine clearance below 60 mL/min
- □ History of myocardial infarction
- □ Hypertension
- □ Do not know /no opinion

15. Asymptomatic people must have an HIV test before starting PrEP:
- □ Yes
- □ No
- □ Do not know /no opinion

16. STDs must be ruled out before starting PrEP:
- □ Yes
- □ No
- □ Do not know /no opinion

17. While taking PrEP, people must have regular clinical and analytical follow-up visits with the healthcare provider every:
- □ Week.
- □ Month.
- □ 3 months.
- □ Year.
- □ Do not know /no opinion.

18. PrEP reduces the risk of getting HIV and other sexually transmitted diseases (STDs):
- □ Yes
- □ No
- □ Do not know /no opinion

THIRD SECTION: ATTITUDES TOWARDS PrEP

Please, choose the option below that best describes your opinion.

	Strongly disagree	Disagree	Neither agree nor disagree	Agree	Strongly agree
19. There is insufficient evidence at this time to consider PrEP an appropriate prevention strategy					
20. PrEP is a cost-effective HIV prevention intervention if used with an appropriate population of patients					
21. PrEP will cause people to have more risky sex					
22. People with very high risk of HIV infection must be encouraged to use condoms rather than to take PrEP					
23. PrEP may be given preference over other preventive strategies					
24. Widespread use of PrEP will likely significantly increase rates of antiretroviral resistance					
25. PrEP is associated to important side effects					
26. PrEP adherence is critical to efficacy					
27. Nurses play an important role in prevention of sexual risk behaviors and STDs, such as					

HIV, through patient education					
28. It is neccessary to allocate resources for PrEP research					
29. PrEP must be paid by the patient					
30. PrEP must be financed by the Social Security System					
31. PrEP is recommended for:					
a. Men who have sex with men					
b. Heterosexual people					
c. Transgender people					
d. Sex workers					
e. People with multiple sex partners					
f. People with history of STD					
g. Injection drug users					
h. Serodiscordant couples					
32. PrEP prescription remains suboptimal because...					
a. It is difficult to identify the target population					
b. Physicians have insufficient knowledge of PrEP					
c. There is lack of time to follow-up the patients					
d. There is lack of time to advise on the prevention of sexual risk behaviors					
e. There are not protocols or clinical practice guidelines					

f. Healthcare professionals do not discuss sexual risk behaviors with their patients					
33. PrEP demand is not higher because…					
a. Its existence is unknown					
b. People do not know where it can be obtained					
c. It could stigmatize people					
d. People do not know its efficacy					

FOURTH SECTION: INTEREST IN PrEP

34. What is your level of interest in learning more about PrEP for HIV prevention?

- □ Not interested at all
- □ Not interested
- □ Neutral
- □ Interested
- □ Very interested

35. Please, answer only if your previous answer was Interested/Very interested. How would you like to learn more about PrEP? (Please, choose all that apply):

- □ Textbooks
- □ Subject of the training programme in the nursing degree
- □ Clinical practice guidelines
- □ Online methods
- □ Other:________________

36.When do you think the nursing degree should spend more time training on PrEP? (Choose all that apply):

- □ 1º
- □ 2º
- □ 3º
- □ 4º
- □ None of the above

FIFTH SECTION: INTENTIONS

Before continuing, you need to know the following information about PrEP:

PrEP is a preventive intervention that reduces the risk of HIV infection in HIV-seronegative people at high risk of infection. The authorized combination is based on tenofovir disoproxil fumarate (TDF) plus emtricitabine (FTC) in a single tablet (Truvada®), once daily. In combination with other preventive strategies, it is highly effective when administered before exposure to HIV only if there is good therapeutic adherence.

PrEP does not protect against other STDs, so other protection strategies such as condom use, are necessary during sexual intercourse.

Before starting PrEP, HIV and other STDs must be ruled out, and it is necessary to assess creatinine clearance. Likewise, while taking PrEP, people must have regular clinical and analytical follow-up visits with the healthcare provider every 3 months.

The main side effects of PrEP are: gastrointestinal symptoms, nausea, headache, loss of appetite or tiredness. They generally subside over time and are not life threatening.

According to the information previously provided, if you belonged to a population group at high risk for HIV infection, would you be willing to receive PrEP?
□ Yes<br>□ No<br>□ Do not know /no opinion

Table S3. Recoding into two categories (correct and incorrect) of the answers to the questions included in the knowledge section of the questionnaire.

**Questions**	**Answers dichotomized**
Q11. The antiretroviral drugs used for PrEP approved by the FDA are:	
Emtricitabine + tenofovir (Truvada®)	Correct
Lopinavir (Kaletra®)	Incorrect
Emtricitabine + tenofovir + efavirenz (Atripla®)	
DK/NO	
Q12. The PrEP is administered by:	
Oral route	Correct
Intravenous route	Incorrect
Subcutaneous route	
Intramuscular route	
DK/NO	
Q13. According to the FDA, the antiretroviral drugs used for PrEP must be taken:	
Daily	Correct
Weekly	Incorrect
Before sexual intercourse	
After sexual intercourse	
DK/NO	
Q14. PrEP is contraindicated in patients with:	
Creatinine clearance below 60 mL/min	Correct
History of myocardial infarction	Incorrect
Hypertension	
DK/NO	
Q15. Asymptomatic people must have an HIV test before starting PrEP:	
Yes	Correct
No	Incorrect
DK/NO	
Q16. STDs must be ruled out before starting PrEP:	
Yes	Correct
No	Incorrect
DK/NO	
Q17. While taking PrEP, people must have regular clinical and analytical follow-up visits with the healthcare provider every:	
3 months	Correct
Week	Incorrect
Month	
Year	
DK/NO	
Q18. PrEP reduces the risk of getting HIV and other STDs:	
No	Correct
Yes	Incorrect
DK/NO	

Abbreviations: DK/NO. Do not know/no opinion; FDA. Food and Drug Administration; HIV. Human immunodeficiency virus; PrEP. Pre-exposure prophylaxis; Q. Question; STDs. Sexually transmitted diseases.

Table S4. Overall knowledge score according to the sex, age, class year and prior training or knowledge of HIV/AIDS and PrEP.

		Overall knowledge score	
		Med (IQR)	p
Sex	Male	1 (0-2)	0.283
	Female	1 (0-2)	
Age	18-19 years	1 (0-2)	0.409
	≥ 20 years	1 (0-2)	
Class year	First year	0 (0-2)	0.008
	Second year	1 (0-2)	
	Third year	1 (0-3)	
	Fourth year	2 (0-3)	
Prior training on HIV / AIDS	Yes	1 (0-2)	0.614
	No	1 (0-2)	
	DK/NO	0 (0-2,75)	
Prior training on PrEP	Yes	1 (0-3)	0.047
	No	0 (0-2)	
	DK/NO	3 (1-4)	

The overall knowledge scores were compared according to the sex, age, class year and prior training or knowledge of HIV/AIDS and PrEP. Statistical significance ($p < 0.05$) was determined by Kruskal-Wallis and Mann-Whitney U tests. Abbreviations. AIDS. Acquired immunodeficiency syndrome; DK/NO. Do not know/no opinion; HIV. Human immunodeficiency virus; IQR. Interquartile range; Med. Median; PrEP. Pre-exposure prophylaxis.

Table S5. Bivariate logistic regression analysis for attitudes about pre-exposure prophylaxis (PrEP).

Question	Sex<br>Female vs male<br>Adjusted OR (95% CI)	Age<br>>19 vs 18-19 years old<br>Adjusted OR (95% CI)	Class year<br>Third-Fourth vs First-Second<br>Adjusted OR (95% CI)	Overall knowledge score<br>Good vs other<br>Adjusted OR (95% CI)
Q19	0.56 (0.21-1.49)	0.97 (0.44-2.10)	0.76 (0.34-1.71)	0.62 (0.21-1.86)
Q20	0.83 (0.35-1.97)	3.02 (1.64-5.57)	3.22 (1.68-6.17)	1.72 (0.76-3.87)
Q21	0.93 (0.40-2.17)	0.90 (0.48-1.69)	1.17 (0.60-2.28)	2.26 (0.99-5.13)
Q22	1.26 (0.42-3.79)	2.15 (0.96-4.86)	2.09 (0.90-4.90)	0.69 (0.22-2.18)
Q23	0.87 (0.34-2.18)	0.66 (0.33-1.32)	0.71 (0.33-1.52)	1.25 (0.51-3.07)
Q24	0.99 (0.23-4.19)	3.84 (1.06-13.9)	1.85 (0.55-6.23)	0.31 (0.07-1.47)
Q25	1.35 (0.30-6.04)	1.36 (0.43-4.32)	2.84 (0.91-8.89)	2.50 (0.64-9.68)
Q26	0.79 (0.34-1.80)	2.20 (1.21-3.98)	2.40 (1.28-4.49)	1.03 (0.46-2.31)
Q27	0.91 (0.25-3.29)	1.04 (0.43-2.52)	1.50 (0.58-3.88)	1.19 (0.20-7.04)
Q28	1.77 (0.58-5.39)	0.62 (0.31-1.25)	0.54 (0.25-1.18)	1.25 (0.38-4.14)
Q29	0.57 (0.17-1.95)	0.85 (0.39-1.85)	2.03 (0.79-5.19)	0.90 (0.27-2.99)
Q30	1.89 (0.55-6.56)	1.19 (0.54-2.59)	0.43 (0.17-1.10)	1.69 (0.49-5.86)
Q31a	0.31 (0.05-2.18)	0.61 (0.15-2.45)	0.14 (0.03-0.70)	0.45 (0.05-3.81)
Q31b	2.29 (0.44-12.00)	0.66 (0.15-2.84)	0.97 (0.21-4.47)	0.83 (0.12-5.64)
Q31c	0.35 (0.07-1.69)	1.57 (0.50-4.93)	2.99 (0.84-10.65)	5.50 (0.96-31.68)
Q31d	1.22 (0.24-6.23)	0.35 (0.12-1.05)	0.27 (0.08-0.9)	2.38 (0.45-2.72)
Q31e	1.27 (0.21-7.50)	2.75 (0.88-8.63)	1.74 (0.46-6.6)	0.14 (0.03-0.69)
Q31f	1.1 (0.25-4.93)	0.54 (0.19-1.51)	0.65 (0.21-2.05)	0.29 (0.07-1.13)
Q31g	0.38 (0.08-1.92)	2.85 (1.02-7.92)	6.22 (1.73-22.38)	1.51 (0.35-6.60)
Q31h	2.98 (0.61-14.47)	0.68 (0.24-1.90)	0.78 (0.24-2.61)	2.34 (0.53-10.33)
Q32a	1.04 (0.39-2.80)	0.68 (0.33-1.40)	0.75 (0.35-1.63)	0.44 (0.16-1.20)
Q32b	2.05 (0.63-6.68)	1.42 (0.62-3.26)	1.41 (0.58-3.44)	0.76 (0.23-2.51)
Q32c	0.5 (0.14-1.71)	1.18 (0.48-2.93)	0.86 (0.33-2.22)	1.49 (0.45-4.98)
Q32d	1.59 (0.55-4.62)	0.59 (0.26-1.33)	0.80 (0.35-1.84)	1.01 (0.36-2.88)
Q32e	3.54 (0.40-31.2)	0.64 (0.21-1.93)	0.44 (0.12-1.64)	0.24 (0.03-2.13)

Table S5. *Cont.*

Q32f	0.63 (0.20-1.95)	1.75 (0.77-3.96)	1.76 (0.73-4.26)	1.68 (0.54-5.22)
Q33a	0.91 (0.26-3.2)	0.36 (0.14-0.92)	0.15 (0.04-0.52)	0.91 (0.23-3.60)
Q33b	1.38 (0.32-5.93)	2.76 (0.92-8.29)	2.20 (0.62-7.84)	1.09 (0.24-4.94)
Q33c	1.13 (0.37-3.41)	0.88 (0.40-1.90)	1.01 (0.44-2.32)	2.57 (0.75-8.75)
Q33d	0.83 (0.20-3.37)	0.58 (0.22-1.51)	2.79 (0.91-8.58)	0.84 (0.20-3.58)

The name of questions (Q) can be found in Table 3. Abbreviations. CI. Confidence interval; OR. Odds ratio.
